# Design and characterization of an eight‐element passively fed meander‐dipole array with improved specific absorption rate efficiency for 7 T body imaging

**DOI:** 10.1002/nbm.4106

**Published:** 2019-05-27

**Authors:** Irena Zivkovic, Catalina Arteaga de Castro, Andrew Webb

**Affiliations:** ^1^ C.J. Gorter Center for High Field MRI, Department of Radiology Leiden University Medical Center Leiden The Netherlands; ^2^ Imaging Division University Medical Centre Utrecht Utrecht The Netherlands

**Keywords:** 7 T MRI coils, dipole antennas, passive feed, specific absorption rate, transmit efficiency

## Abstract

**Objective:**

To evaluate the transmit efficiency and specific absorption rate (SAR) efficiency of a new eight‐element passively fed meander‐dipole antenna array designed for body MRI at 7 T, and to compare these values with a conventional directly fed meander‐dipole array.

**Methods:**

The main radiating element of the passively fed dipole is printed on one side of a dielectric substrate and is capacitively coupled to a shorter feeding element (connected to the coaxial cable) printed on the opposite side of the substrate. The transmit (B_1_^+^) field and SAR were simulated on a phantom and on a human voxel model for both a passively fed and a directly fed single element. Two eight‐channel arrays containing, respectively, directly and passively fed meander dipoles were then simulated, and experimental B_1_^+^ maps and T_2_‐weighted spin echo images of the prostate were obtained in vivo for four healthy volunteers.

**Results:**

In simulations, the mean transmit efficiency (B_1_^+^ per square root input power) value in the prostate was ~ 12.5% lower, and the maximum 10 g average SAR was 44% lower for the array containing passively fed dipoles, resulting in ~ 15% higher SAR efficiency for the passively fed array. In vivo RF‐shimmed turbo spin echo images were acquired from both arrays, and showed image SNRs within 5% of one another.

**Conclusion:**

A passive‐feeding network for meander‐dipole antennas has been shown to be a simple method to increase the SAR efficiency of a multi‐element array used for body imaging at high fields. We hypothesize that the main reason for the increase in SAR efficiency is the storage of the strong conservative electric field in the dielectric between the feeding element and the radiating element of the dipole. The passive‐feeding approach can be generalized to other dipole geometries and configurations.

**Abbreviations used**
B_1_^+^: transmit field
DREAM: dual refocusing echo acquisition mode
ɛ_r_: relative permittivity
FoV: field of view
PCB: printed circuit board
PMMA: polymethylmethacrylate
SAR: specific absorption rate
STEAM: stimulated echo acquisition mode
tan δ: loss tangent

## 1 INTRODUCTION

Human MRI at high field strengths is challenging due to significant inhomogeneities in the transmit (B_1_^+^) fields that occur as a result of the relatively short RF wavelength in tissue compared with the dimensions of the human body. This is particularly evident in body applications at field strengths of 7 T and higher,^1^, ^2^ which has led to the use of transmit arrays consisting of multiple (typically 8–32 elements) transmit antennas.^3^, ^4^, ^5^, ^6^, ^7^, ^8^, ^9^ This approach uses individually controlled transmit channel phases and/or magnitudes. A number of different resonators have been used for the individual elements of these arrays, including loops and microstrips,^8^, ^10^, ^11^ but in the past few years various forms of dipole antennas have become the predominant geometry.^12^, ^13^, ^14^, ^15^, ^16^ The intrinsic far‐field nature of a dipole is advantageous in terms of transmitting electromagnetic energy into deep‐lying organs within the body.^17^ Dipoles have been shown both theoretically 18, 19 and experimentally^20^, ^21^, ^22^, ^23^ to be efficient transmit elements for body imaging at 7 T in particular, and have also recently been used at 10.5 T.^24^

After the initial demonstration of the performance of a conventional straight dipole, a substantial amount of research has concentrated on optimizing the dipole geometry in order to improve a combination of the transmit efficiency (B_1_^+^ field per square root input power) and the specific absorption rate (SAR) efficiency (B_1_^+^ field to the square root maximum specific absorption rate [SAR_10g, max_]). For example, the fractionated dipole antenna presented in^21^ is a straight printed dipole with several meandered structures included in each leg of the dipole, which act as inductances to increase the electrical length of the dipole. This design produces the lowest SAR_10g, max_ among current dipoles used for high field imaging. Dipoles can be used as transmit‐only or transceiver elements, either alone or in combination with loop antennas as additional receive elements .^25^ Fractionated dipoles can be combined in pairs to give independent excitation of the even and odd modes of the dipole pair.^26^

For all of the dipole geometries described in the literature, including the meander dipole, the element is directly fed via a partially or fully balanced lumped matching element network consisting of capacitors and/or inductors. However, it is also possible to feed dipole elements passively, ie via inductive and capacitive coupling from a passive structure that is coupled to the dipole.^27^, ^28^ In this study, we evaluate experimentally and in simulations the passive‐feeding approach for a 300 mm long meander‐dipole antenna used as an array element for prostate imaging at 7 T. The main radiating antenna element was printed on one side of a dielectric substrate, coupled to a shorter passive feeding element printed on the opposite side. Numerical and experimental comparisons were performed with a conventional array containing directly fed meander‐dipole antennas.

## 2 MATERIALS AND METHODS

### 2.1 Dipole fabrication

**Figure 1:**
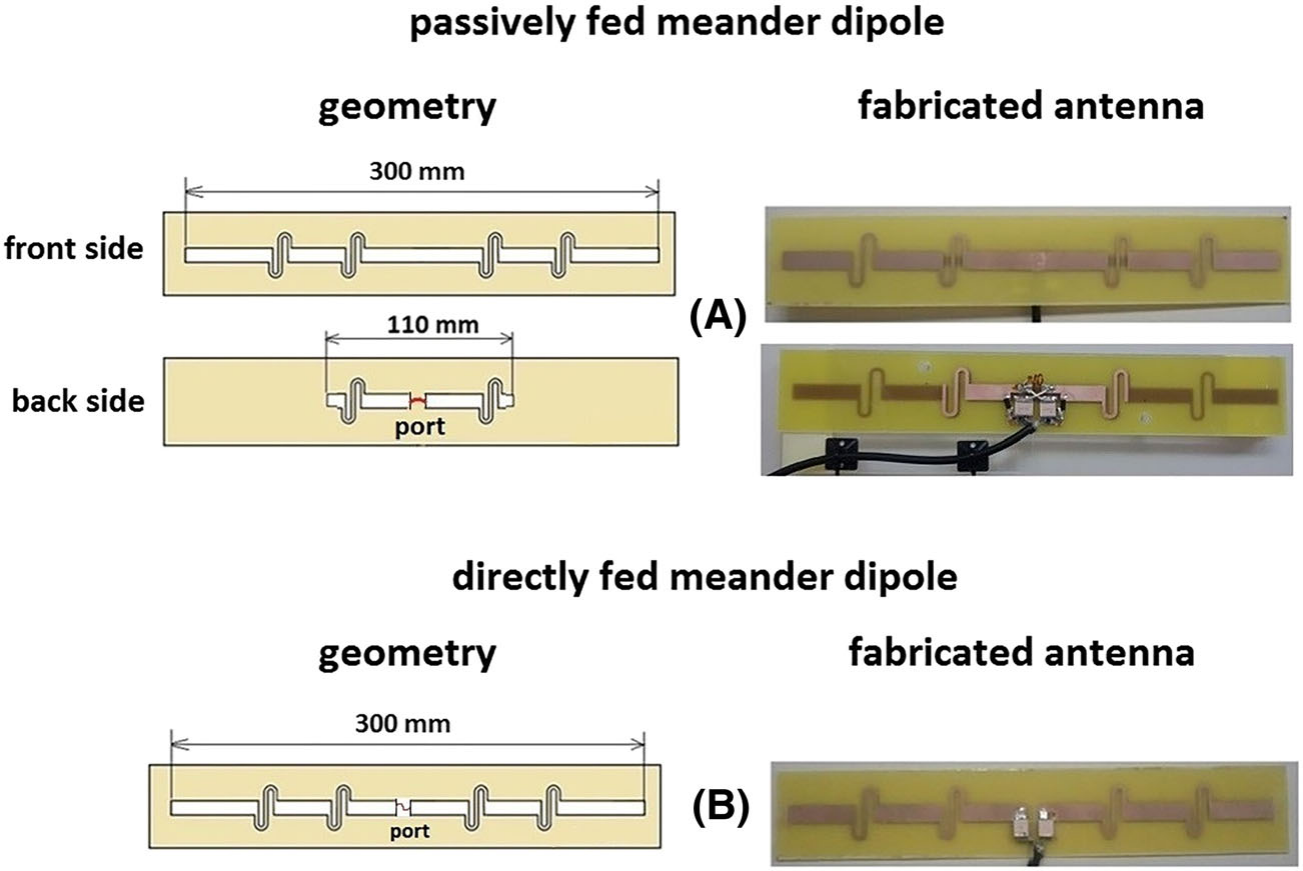
Designed geometry and photos of fabricated meander‐dipole antennas with (A) passive feed and (B) direct feed

The passively and directly fed meander‐dipole geometries are shown in Figure 1. The length and geometry of the short feeding dipole (in the passively fed meander‐dipole design) was chosen via a parametric study (data not shown), in which the length of the feeding dipole was varied from 90 to 150 mm, in steps of 10 mm, and both a straight and meandered geometry were studied, in order to obtain the maximum SAR efficiency. The design of the directly fed meander dipole is identical to that described previously.^21^ The dipoles were each 300 mm long and were fabricated on an FR‐4 printed circuit board (PCB) substrate (ɛ_r_ = 4.3, tan δ = 0.025, substrate thickness = 1.5 mm). For a passively fed dipole (Figure 1A), one side of the PCB (“back” side) contained a short feeding element (length = 110 mm) with a small meander at the end of each leg. The other side of the PCB (“front” side) contained a larger radiating dipole and was oriented towards the phantom/subject. The passively fed meander dipole had a 33 nH inductor connected between the legs of the short feeding element and two identical 3.9 pF matching capacitors (Figure 1A). The directly fed meander dipole (Figure 1B) had two identical matching capacitors with a value of 22 pF. For the transmit/receive array eight directly fed and eight passively fed meander antennas with the same total dimensions as described above were constructed. A 20 mm thick polymethylmethacrylate (PMMA) spacer was attached to the face of the dipole.

S_11_ was measured for both the directly and passively fed meander dipoles as a function of the distance between the single antenna and the phantom: this distance was varied from 20 to 70 mm in steps of 10 mm. The reference point for both antennas was the feed point, ie the short feeding dipole was at the same distance from the phantom as the feed point of the directly fed dipole.

### 2.2 Electromagnetic simulations and antenna measurements

**Figure 2:**
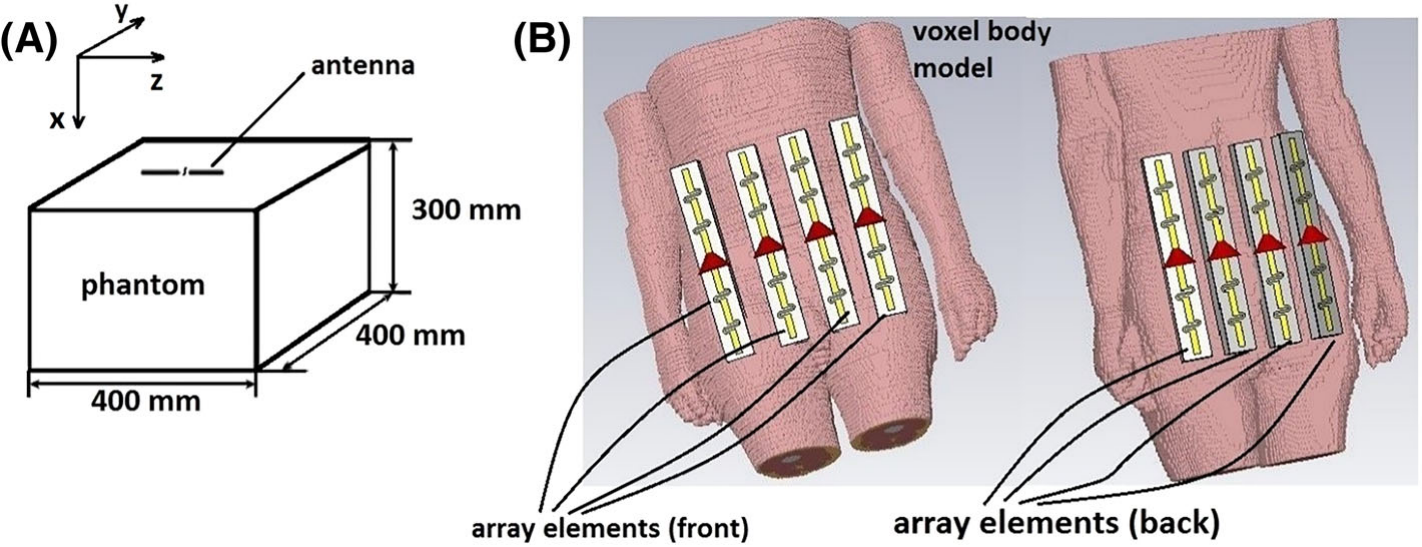
Simulated setups: (A) phantom with a single antenna on top and (B) human body voxel model with eight‐channel dipole array

Electromagnetic simulations were performed in CST Microwave Studio 2016 (CST Studio Suite, Computer Simulation Technology, Darmstadt, Germany). Simulations were first performed with a single antenna element on a square phantom (Figure 2A). Subsequently, eight‐element arrays containing directly or passively fed dipoles (Figure 2B) were simulated using the voxel model Gustav (CST Studio Suite, Computer Simulation Technology). The voxel size in Gustav was 2 x 2 x 2 mm. The PMMA spacer placed between the antenna and phantom/voxel model was included in the simulations. The dielectric properties of the phantom used were ɛ_r_ = 50 and σ = 0.6 S/m. To evaluate the B_1_^+^ and SAR_10g, max_ efficiency of the single antenna, results were normalized to 1 W of accepted power. In the eight‐element body array simulations, B_1_^+^ and SAR_10g, max_ were normalized to 8 W of accepted power. Simulations of the SAR and B_1_^+^ of passively and directly fed dipole antennas were performed with open boundary conditions in all directions. The mesh type used was hexahedral. The total number of cells for the single antenna phantom simulations was 1.5 million cells and the simulation time was around two minutes on a standard desktop computer. The total number of cells for the eight‐channel body array with voxel model Gustav was ~ 14.5 million and the simulation time was around 30 minutes.

Simulated RF shimming was performed by adjusting the phases of the individual channels to produce the maximum constructive interference of all channels in the prostate region. The phase of the B_1_^+^ field produced by each channel in the prostate was estimated, and the corresponding negative phase applied to the transmit channel was such that the phases of all channels were zero in the prostate, and that the B_1_^+^ fields added constructively.

### 2.3 MRI measurements

All studies were approved by the medical ethics committee of the Leiden University Medical Center. In vivo experiments were performed using a Philips (Philips Best, The Netherlands) 7 T whole body scanner equipped with an eight‐channel parallel transmit architecture (Philips Achieva). Single antenna B_1_^+^ maps were measured on a phantom, and in vivo B_1_^+^ maps were obtained using the dual refocusing echo acquisition mode (DREAM)^29^ sequence with the following parameters: field of view (FoV) = 400 x 320 x 25 mm, voxel size = 5 x 5 x 5 mm, slices = 5, tip angle = 10°, stimulated acquisition mode (STEAM) angle = 50°, TE/TR = 1.97/15 ms, one signal average. Based on the in vivo B_1_^+^ maps, phase‐based RF shimming was performed as described above (with a linear optimization algorithm written in MATLAB on a PC linked to the MR scanner) to obtain the maximum B_1_^+^ in the prostate of a healthy volunteer. Equal magnitude excitation was used on all channels.

MRI‐based thermal measurements were performed using the proton reference frequency method^30^ with antennas placed on the phantom. The 3DGRE sequence used for heating had the following parameters: TR/TE = 14/10 ms, flip angle (FA) = 10^0^, scan duration = 1800 s; the final temperature was measured after 30 minutes of scanning.

Four healthy volunteers with different body mass indices (BMIs) were imaged. The BMIs varied from 20.8 to 28.7 kg/m^2^. The sequence parameters used for in vivo imaging were as follows: a T_2_‐weighted turbo spin echo (TSE) sequence, TE/TR = 110/6000 ms, flip angle = 90°, TSE factor = 20, FoV = 250 x 380 x 16 mm, voxel size = 0.8 x 0.8 mm, slice thickness = 3 mm, one signal average. A noise correlation matrix was generated by acquiring data with the RF and gradient channels turned off, with the noise correlation coefficient (*ρ*_*ij*_) defined as

$$\rho_{\mathrm{ij}}=\frac{\mathrm{cov}\left(n_{i}n_{j}\right)}{\sigma_{\mathrm{ni}}\sigma_{\mathrm{nj}}}$$

where *n*_*i*_ is the noise measured in coil element *i*. SNR maps were acquired using the method described previously .^31^

## 3 RESULTS

**Figure 3:**
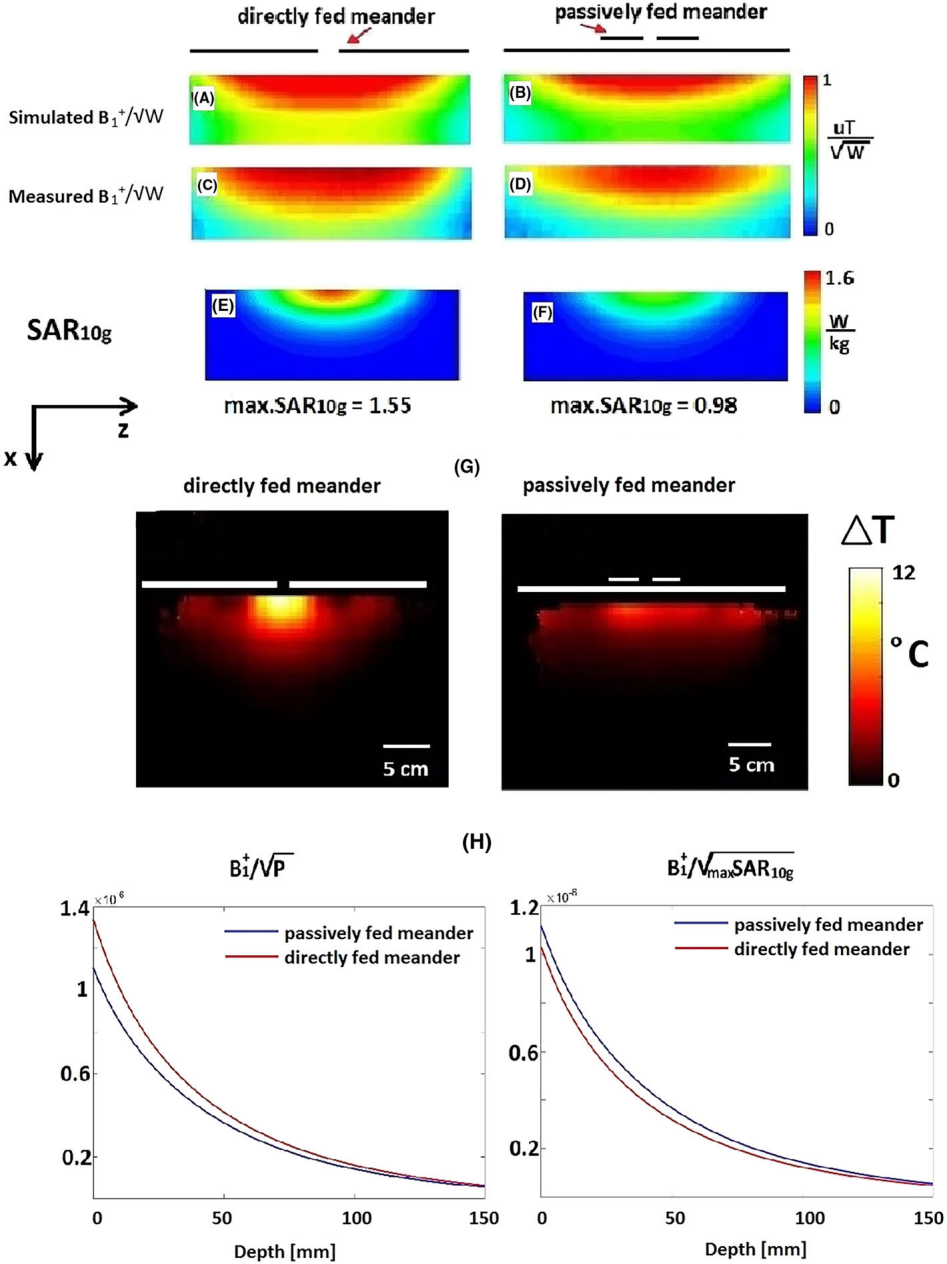
(A, B) Simulated and (C, D) measured B_1_^+^ fields for the single directly and passively fed meander dipoles on a phantom. (E, F) Simulated max SAR_10g_ for the single directly and passively fed meander dipoles on a phantom. (G) Thermometry measurements of (left) directly and (right) passively fed meander dipoles. (H) Simulated (left) B_1_^+^ per square root of power and (right) B_1_^+^ per square root of max SAR_10g_ of both antennas along a central line on a phantom

Figure 3 shows simulated and measured B_1_^+^ and SAR_10g, max_ values, as well as the thermometry measurements from single dipole elements placed on the phantom. For the passively fed meander‐dipole antenna, the simulated and measured B_1_^+^ field (normalized to 1 W of accepted power) of a single element is lower than that of the directly fed antenna (Figure 3A‐D), while the SAR_10g, max_ is reduced by 37% (Figure 3E and 3F). Figure 3G shows the results of the MRI‐based thermal measurements performed for the same input power and the same heating time. The passively fed antenna produced ~ 50% less heating (measured at the point of maximum heating, which was directly under the central point of the dipole) than the directly fed antenna. The maximum temperature increase produced by the directly fed meander antenna was 12°C while the maximum temperature increase for the passively fed meander antenna was 6°C.

Figure 3H shows a graph of the B_1_^+^ per square root of unit power (transmit efficiency) and B_1_^+^ per square root of SAR_10g, max_ (SAR efficiency) for both directly and passively fed dipoles plotted along the central line in the phantom. The figures show that the transmit efficiency is higher for the directly fed dipole at the surface of the phantom, whereas the SAR efficiency is higher for the passively fed antenna for all depths. The reference point for the measurements was the feed point, ie the short feeding dipole was at the same distance from the phantom as the feed point of the directly fed dipole (note that in the case where the radiating conductors are instead used as reference points, the B_1_^+^ efficiency increased by 2.7% at the surface and by less than 0.5% at 5 cm depth for the directly fed meander antenna).

**Figure 4:**
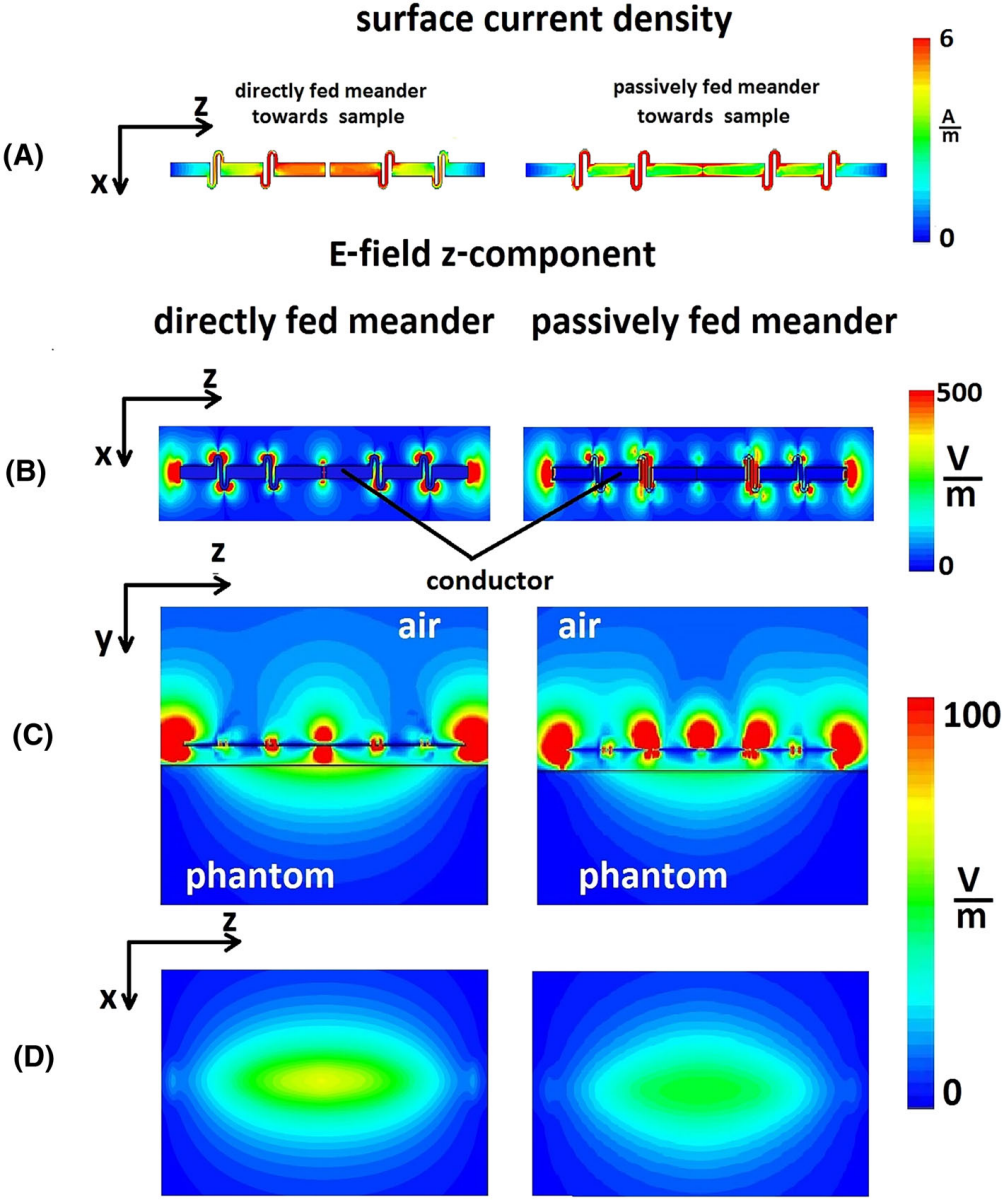
(A) Surface current density on a conductors surface of the (left) directly and (right) passively fed meander dipole. Orientation of the conductors surface was towards a phantom. (B) Z‐component of the E‐field at the surface of the (left) directly and (right) passively fed meander dipole. (C) Side view of the E‐field z‐component of the dipole antenna placed 20 mm away from the phantom. (D) Z‐component of the E‐field at the surface of the phantom of the (left) directly and (right) passively fed meander dipole

In order to investigate the origin of the increased SAR efficiency, Figure 4A shows simulated surface current distribution on an antenna's conductor facing a phantom of (left) directly and (right) passively fed meander dipole. Figure 4B shows the simulated z‐component of the E‐field (E_z_) on the surface of the dipoles. For both cases, the strongest E_z_ values were at the feed point, around the meanders and at the end of the dipoles. Figure 4C shows the E_z_ field from a side view of the simulated setup containing a directly/passively fed meander‐dipole antenna placed on a 20 mm spacer above the phantom. Figure 4D shows the E_z_ field at the surface of the phantom (top view). The E_z_ field component was higher for the directly than for the passively fed dipole throughout the entire phantom.

**Figure 5:**
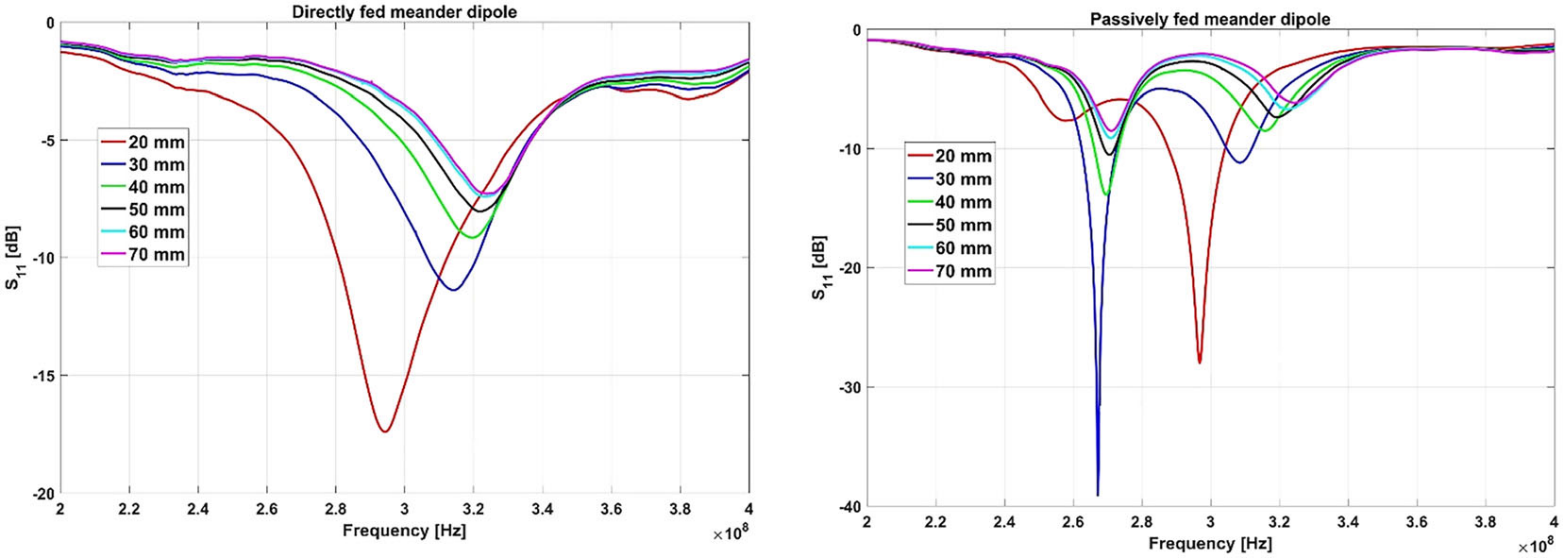
Measured S_11_ of the (left) directly and (right) passively fed meander dipole when the distance from the phantom has been varied

Figure 5 shows the measured S_11_ of both dipole antennas as a function of the distance from the phantom, varying from 20 to 70 mm in steps of 10 mm. Both antennas were impedance‐matched for a 20 mm distance from the phantom. As the distance increased, the S_11_ minima of both antennas shifted towards higher frequencies by the same amount, indicating an equal amount of inductive coupling to the sample (note that the S_11_ plot of the passively fed antenna has two peaks: one from the long passive element and the other from the short feeding dipole).

**Figure 6:**
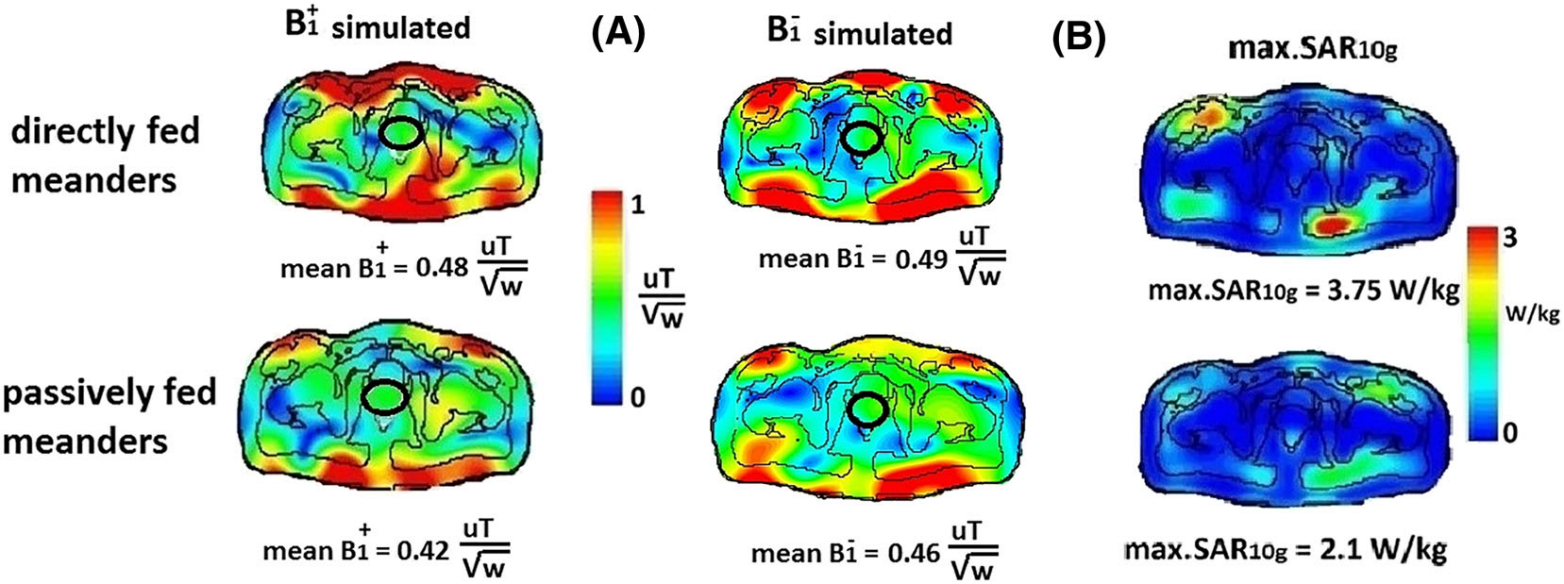
(A) Simulated (left) B_1_^+^ and (right) B_1_^−^ per square root of power efficiency (normalized to 8 W of accepted power) and (B) simulated max SAR_10g_ (normalized to 8 W of accepted power). Optimal phases of channels were determined for a maximum constructive B_1_^+^ in prostate

Figure 6 shows the simulated B_1_^+^ and B_1_^−^ fields and SAR_10g, max_ values for the two different eight‐channel arrays (both passively and directly fed antennas) on the body voxel model. The simulated B_1_^+^ field was ~ 12.5% lower in the prostate region for the passively fed meander‐dipole arrays while the B_1_^−^ field was ~ 6% lower for the passively than the directly fed meander. The simulated SAR_10g, max_ produced by the passively fed dipole array is 44% lower than that from the directly fed dipole array, giving an increase in SAR efficiency of ~ 15%.

**Figure 7:**
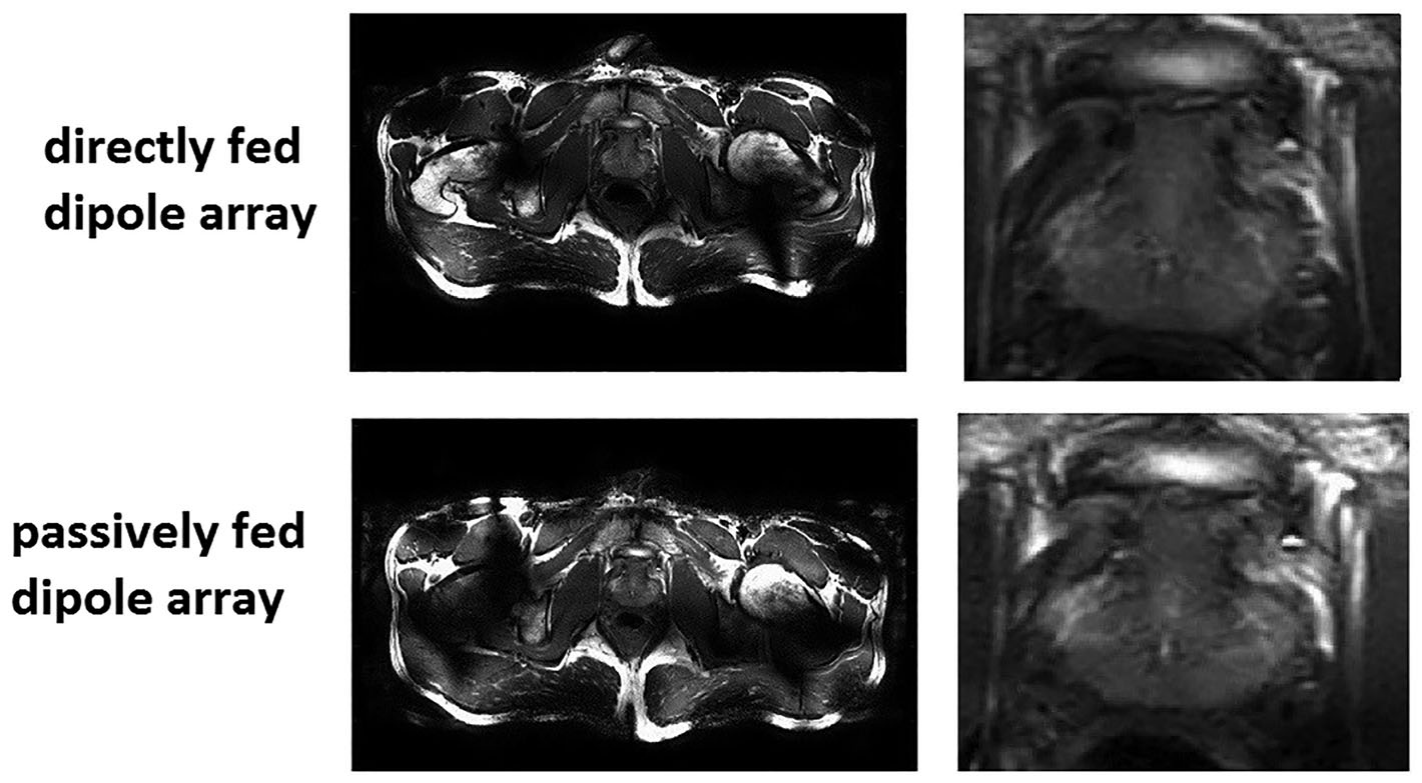
In vivo TSE prostate images made with eight‐channel body arrays of (upper row) directly fed and (lower row) passively fed meander dipoles with zoomed prostate images

**Figure 8:**
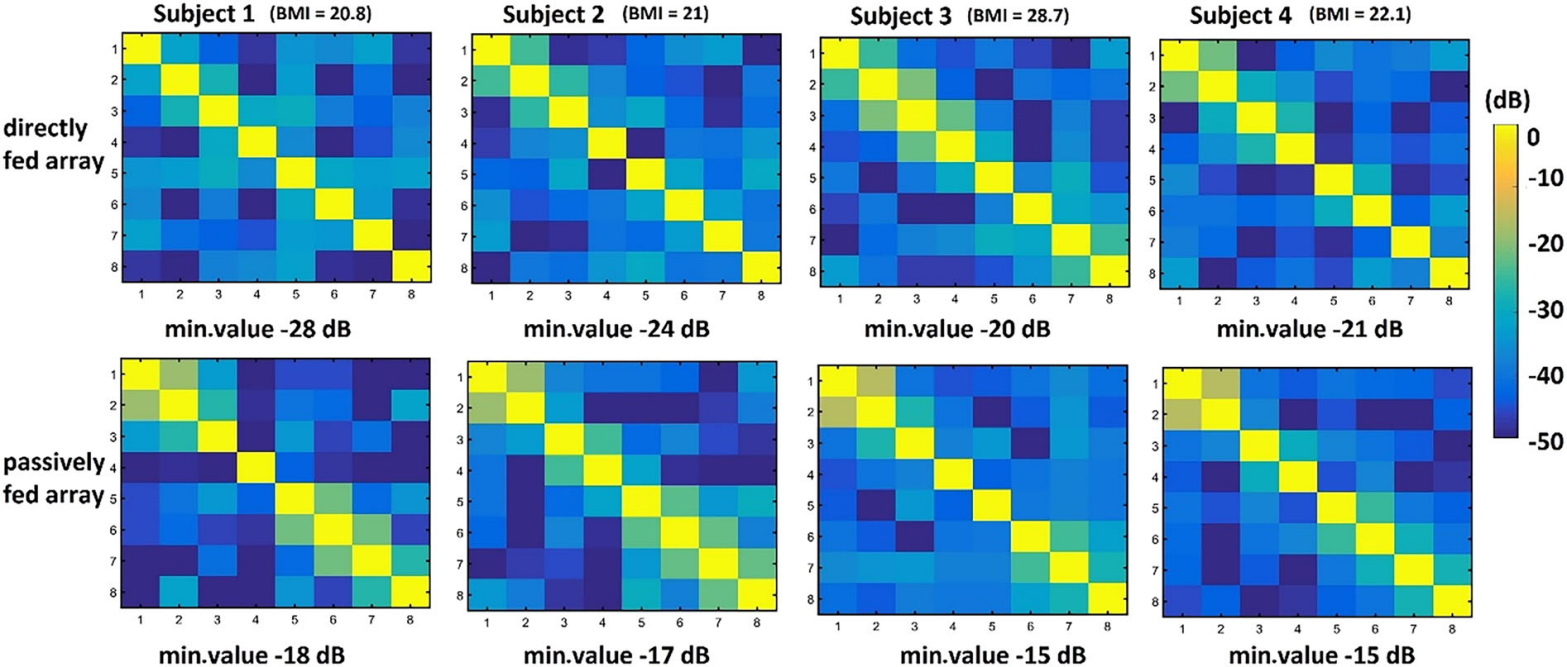
Noise correlation matrices of four volunteers scanned with (upper row) directly fed and (lower row) passively fed array. The body mass indices of volunteers varied from 20.8 to 28.7 kg/m^2^

Figure 7 shows in vivo images from one of the four healthy volunteers. The experimentally (in vivo) measured noise correlation matrices of both arrays and for all four volunteers are shown in Figure 8. The coupling coefficients between individual channels of both arrays were below −15 dB. The measured averaged B_1_^+^ field in the prostate region was ~ 15% lower, while the averaged SNR in a prostate was ~ 5% lower for the array containing passively fed dipoles.

Figure 8 shows measured noise correlation matrices of four volunteers with BMIs spanning from 20.8 to 28.7 kg/m^2^. In cases of both passively and directly fed arrays, the highest coupling coefficient was −15 dB. The highest inter‐element coupling corresponded to the subject with the highest BMI value, which can be explained by the fact that tuning and matching of antennas were performed on a homogeneous phantom, where the antennas were placed at a 20 mm distance from the phantom, and for this particular volunteer the effective distance was much greater than this distance.

## 4 DISCUSSION

This study compares the transmit and SAR efficiencies of two identical meander‐dipole configurations, the difference being that one is passively fed and the other is directly fed. The results show that the transmit efficiency of a passively fed dipole or dipole array is reduced by ~ 15% at penetration depths of ~ 10–15 cm, whereas the SAR_10g, max_ is reduced by more than 40% and the SAR efficiency is increased by ~ 15%. Because most high field body imaging applications are limited by the SAR rather than the absolute power available from the multiple RF amplifiers, these results suggest that changing from an actively fed to a passively fed architecture could have significant benefits for body imaging at high fields.

Why does a passive feed reduce the SAR and increase the SAR efficiency? In order to reduce SAR the total electric field penetrating the sample should be minimized, ideally without significantly affecting the magnetic B_1_^+^ field component. The SAR depends on both the conservative and inductive (nonconservative) electric field components. The conservative electric field originates from a gradient in charge accumulation on the antenna's conductor. From Figure 4B and 4C, we conclude that the highest E_z_ components (which can be attributed to the conservative electric field) occur at the location of the meanders, at the feed point, and at the end of the antenna's legs. The E_z_ component is important because, based on the boundary conditions at the surface of the phantom, the tangential component of the electric field in the air and in the phantom must be the same. The main attribute of a passive‐feed network is that it provides partial shielding of the E_z_ component of an electric field in a driven device. Figure 4C shows that the strongest E_z_ field of a directly fed antenna originates from the middle (feed) point of the dipole. In the case of the passively fed dipole, the middle point of the feed is “shielded” from the sample by the passive element and the induced E_z_ field in the phantom is reduced. From the surface current distribution at the antenna's conductor facing the phantom (Figure 4A), the maximum current distribution for the directly fed meander is in the central section of the antenna. The magnitude of the surface current distribution for the passively fed meander is lower in value and more evenly distributed along the conductor. Therefore, the inductive electric field, related to the surface current distribution on the antenna's radiating conductor, is lower for the passively fed meander. To confirm the hypothesis that the total electric field in a sample is a combination of both conservative and inductive components, we placed a small shielding strip below a conventional actively fed dipole. We observed that the reductions in SAR were not as effective as those with the proposed passive‐feed concept.

The inter‐element coupling of the passively fed dipole (−15 to −18 dB) was found to be slightly less sensitive to in vivo loading conditions than that of the directly fed dipole (−20 to −28 dB), as reflected by the noise correlation coefficients shown in Figure 7, although the absolute values were higher. Again, these results can be explained in terms of the partial shielding effect of the passive element, which reduces slightly the coupling to the sample.

The actual in vivo SAR value depends on the specific phases applied to the individual channels. In this study, we presented simulated SAR values of directly and passively fed dipole arrays where the phases are adjusted to produce the maximum constructive interference in a prostate region. One question to be addressed in future research is whether or not the directly fed dipole antennas would be able to reach a similar SAR efficiency with a different phase setting if some of the superior B_1_^+^ efficiency were to be traded against avoiding local maxima in SAR.

## 5 CONCLUSIONS

This study has shown that the SAR efficiency of a dipole element used for high field MRI can be increased by using a passive‐ rather than an active‐feeding network. Although the results shown here are specifically for a meander dipole, the passive‐feed network approach can also be applied to different dipole geometries. For example, we have performed simulations on the SAR efficiency of a passively fed straight dipole and found similar results to those presented here in terms of the improvement in SAR efficiency. We anticipate that similar behaviour would be found for other types of dipole such as folded,^19^ snake,^32^ or the recently introduced dual mode.^26^ We also anticipate that a passive‐feeding network would increase the SAR efficiency of electrically short dipoles, which are known to have high transmit efficiency but relatively low SAR efficiency, and are suitable, for example, for cardiac or pancreatic imaging.

## References

[1] Vaughan JT , Snyder CJ , DelaBarre LJ , et al. Whole‐body imaging at 7 T: preliminary results. Magn Reson Med. 2009;61:244‐248. 19097214 10.1002/mrm.21751 PMC2875945

[2] Bergen BV , Berg CV , Bartels L , Lagendijk J . 7 T body MRI: B1 shimming with simultaneous SAR reduction. Phys Med Biol. 2007;52:5429‐5441. 17762096 10.1088/0031-9155/52/17/022

[3] Adriany G , van de Moortele PF , Wiesinger F , et al. Transmit and receive transmission line arrays for 7 Tesla parallel imaging. Magn Reson Med. 2005;53:434‐445. 15678527 10.1002/mrm.20321

[4] Brink WM , Gulani V , Webb AG . Clinical applications of dual‐channel transmit MRI: A review. J Magn Reson Imaging. 2015;42:855‐869. 10.1002/jmri.24791. PubMed PMID: 25854179. 25854179

[5] Webb A , Collins C . Parallel transmit and receive technology in high‐field magnetic resonance neuroimaging. Int J Imaging Syst Technol. 2010;20:2‐13.

[6] Katscher U , Bornert P . Parallel RF transmission in MRI. NMR Biomed. 2006;19:393‐400. 16705630 10.1002/nbm.1049

[7] Abraham R , Ibrahim TS . Proposed radiofrequency phased‐array excitation scheme for homogenous and localized 7‐Tesla whole‐body imaging based on full‐wave numerical simulations. Magn Reson Med. 2007;57:235‐242. 17260366 10.1002/mrm.21139

[8] Vaughan JT , Adriany G , Snyder CJ , et al. Efficient high‐frequency body coil for high‐field MRI. Magn Reson Med. 2004;52:851‐859. 15389967 10.1002/mrm.20177

[9] Graessl A , Renz W , Hezel F , et al. Modular 32‐channel transciever coil array for cardiac MRI at 7.0 T. Magn Reson Med. 2014;72:276‐290. 23904404 10.1002/mrm.24903

[10] Zhu YD . Parallel excitation with an array of transmit coils. Magn Reson Med. 2004;51:775‐784. 15065251 10.1002/mrm.20011

[11] Wu B , Zhang X , Wang C , et al. Flexible transceiver array for ultrahigh field human MR imaging. Magn Reson Med. 2012;68:1332‐1338. 22246803 10.1002/mrm.24121 PMC3350759

[12] Lakshmanan K, Cloos M, Lattanzi R, Sodickson D, Wiggins G (editors). The loopole antenna: capturing magnetic and electric dipole fields with a single structure to improve transmit and receive performance. Proceedings of the 22nd Annual Meeting of ISMRM, Milan, Italy; 2014.

[13] Lakshmanan K, Cloos M, Lattanzi R, Sodickson D, Novivkov D, Wiggins G (editors). The circular dipole. Proceedings of the 22nd Annual Meeting of ISMRM, Milan, Italy; 2014.

[14] Chen G, Wiggins G, Sodickson D (editors). 3D curved electric dipole antenna for propagation delay compensation. Proceedings of the 23rd Annual Meeting of ISMRM, Toronto, Canada; 2015.

[15] Duan Q , Nair G , Gudino N , et al. A 7 T spine array based on electric dipole transmitters. Magn Reson Med. 2015;74:1189‐1197. 26190585 10.1002/mrm.25817 PMC4575257

[16] Zhang B, Cloos M, Cheng G, Wiggins G (editors). A size‐adaptable electric dipole array for 7 T body imaging. Proceedings of the 24th Annual Meeting of ISMRM, Singapore; 2016.

[17] Raaijmakers AJ , Ipek O , Klomp DW , et al. Design of a radiative surface coil array element at 7 T: the single‐side adapted dipole antenna. Magn Reson Med. 2011;66:1488‐1497. 21630342 10.1002/mrm.22886

[18] Lattanzi R , Sodickson D . Ideal current patterns yielding optimal SNR and SAR in magnetic resonance imaging: computational methods and physical insights. Magn Reson Med. 2012;68:286‐304. 22127735 10.1002/mrm.23198 PMC3374920

[19] Wiggins G, Zhang B, Lattanzi R, Chen G, Sodickson D (Eds). The electric dipole array: an attempt to match the ideal current pattern for central SNR at 7 Tesla. Melbourne, Australia: ISMRM; 2012.

[20] Winter L , Ozerdem C , Hoffmann W , et al. Design and evaluation of a hybrid radiofrequency applicator for magnetic rsonance imaging and RF induced hyperthermia: electromagnetic field simulations up to 14.0 tesla and proof‐of‐concept at 7.0 Tesla. PLOS ONE. 2013;8:e61661. 23613896 10.1371/journal.pone.0061661 PMC3632575

[21] Raaijmakers A , Italiander M , Voogt I , et al. The fractionated dipole antenna: a new antenna for body imaging at 7 T. Magn Reson Med. 2016;75:1366‐1374. 25939890 10.1002/mrm.25596

[22] Oezerdem C , Winter L , Graessl A , et al. 16‐channel bow tie antenna transceiver array for cardiac MR at 7 tesla. Magn Reson Med. 2016;75:2553‐2565. 26183320 10.1002/mrm.25840

[23] Raaijmakers A , Luijten P , Berg CV . Dipole antennas for ultrahigh‐field body imaging: a comparison with loop coils. NMR Biomed. 2016;29:1122‐1130. 26278544 10.1002/nbm.3356

[24] Erturk M , Wu X , Eryaman Y , et al. Towards imaging the body at 10.5 Tesla. Magn Reson Med. 2017;77:434‐443. 27770469 10.1002/mrm.26487 PMC5191924

[25] Erturk M , Raaijmakers A , Adriany G , Ugurbil K , Metzger G . A 16‐channel combined loop‐dipole transceiver array for 7 tesla body MRI. Magn Reson Med. 2017;77:884‐894. 26887533 10.1002/mrm.26153 PMC4988950

[26] Solomakha G , Leeuwen CV , Raaijmakers A , et al. The dual‐mode dipole: A new array element for 7 T body imaging with reduced SAR. Magn Reson Med. 2018;81:1459‐1469. 30226636 10.1002/mrm.27485

[27] Zivkovic I , O'Reilly T , Brink W , Webb A . Design of a passive feed network to increase the transmit efficiency of dipoles at 7 Tesla. Joint Annual Meeting ISMRM‐ESMRMB, Paris; 2018.

[28] Zivkovic I , O'Reilly T , Brink W , Webb A . Evaluation of Egyptian axe dipole antenna as an array element for head imaging at 7 T MRI. Joint Annual Meeting ISMRM‐ESMRMB; 2018.

[29] Nehrke K , Bornert P . DREAM ‐ a novel approach for robust, ultrafast, multislice B‐1 mapping. Magn Reson Med. 2012;68:1517‐1526. 22252850 10.1002/mrm.24158

[30] De Poorter J , De Wagter C , De Deene Y , Thomsen C , Stahlberg F , Achten E . Noninvasive MRI Thermometry with the Proton Resonance Frequency (PRF) Method: In Vivo Results in Human Muscle. Magn Reson Med. 1995;33:74‐81. 7891538 10.1002/mrm.1910330111

[31] Kelmann P , McVeigh E . Image reconstruction in SNR units: A general method for SNR measurements. Magn Reson Med. 2005;54:1439‐1447. 16261576 10.1002/mrm.20713 PMC2570032

[32] Steensma B, Andrade A, Klomp D, Berg CVd, Luijten P, Raaijmakers A (editors). Body imaging at 7 Tesla with much lower SAR levels: an introduction of the snake antenna array. Proceedings ISMRM, Milan, Italy; 2016.

